# Optimizing Thermoelectric Properties through Compositional Engineering in Ag-Deficient AgSbTe_2_ Synthesized by Arc Melting

**DOI:** 10.1021/acsaelm.3c01653

**Published:** 2024-03-05

**Authors:** Jesús Prado-Gonjal, Elena García-Calvo, Javier Gainza, Oscar J. Durá, Catherine Dejoie, Norbert M. Nemes, José Luis Martínez, José Antonio Alonso, Federico Serrano-Sánchez

**Affiliations:** † Departamento de Química Inorgánica, Universidad Complutense de Madrid, Ciudad Universitaria s/n, Madrid E-28040, Spain; ‡ Instituto de Ciencia de Materiales de Madrid, CSIC, Cantoblanco, Madrid E-28049, Spain; § Departamento de Física Aplicada, Universidad de Castilla-La Mancha, E-13071 Ciudad Real, Spain; ∥ European Synchrotron Radiation Facility (ESRF), 71 Avenue des Martyrs, 38000 Grenoble, France; ⊥ GFMC, Departamento de Física de Materiales, Universidad Complutense de Madrid, Madrid E-28040, Spain

**Keywords:** thermoelectrics, AgSbTe_2_, arc-melting, vacancy, compositional engineering, synchrotron X-ray diffraction, atomic displacement parameters, thermoelectric properties

## Abstract

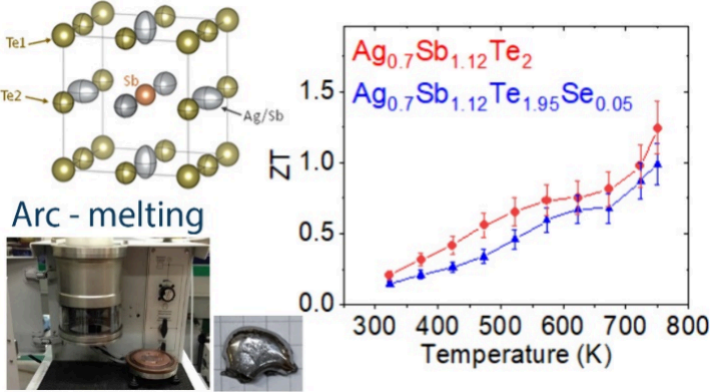

Thermoelectric materials offer a promising avenue for energy management, directly converting heat into electrical energy. Among them, AgSbTe_2_ has gained significant attention and continues to be a subject of research at further improving its thermoelectric performance and expanding its practical applications. This study focuses on Ag-deficient Ag_0.7_Sb_1.12_Te_2_ and Ag_0.7_Sb_1.12_Te_1.95_Se_0.05_ materials, examining the impact of compositional engineering within the AgSbTe_2_ thermoelectric system. These materials have been rapidly synthesized using an arc-melting technique, resulting in the production of dense nanostructured pellets. Detailed analysis through scanning electron microscopy (SEM) reveals the presence of a layered nanostructure, which significantly influences the thermoelectric properties of these materials. Synchrotron X-ray diffraction reveals significant changes in the lattice parameters and atomic displacement parameters (ADPs) that suggest a weakening of bond order in the structure. The thermoelectric characterization highlights the enhanced power factor of Ag-deficient materials that, combined with the low glass-like thermal conductivity, results in a significant improvement in the figure of merit, achieving **zT** values of 1.25 in Ag_0.7_Sb_1.12_Te_2_ and 1.01 in Ag_0.7_Sb_1.12_Te_1.95_Se_0.05_ at 750 K.

## Introduction

Ever-growing energy demands require sustainable, efficient energy solutions fit for the new developments. Thermoelectric (TE) materials offer an alternative way of energy production and thermal expenditure by the direct conversion of heat to electrical energy. This opens up reliable applications for waste heat recovery and localized cooling. Elaboration of functionalized devices is conditioned by the performance of the TE materials, determined by their figure of merit (**zT**): , where *T* is the absolute temperature, σ is the electronic conductivity, κ is the total thermal conductivity, and *S* is the Seebeck coefficient. These usually antagonistic properties depend on the electronic and crystalline structure of the material and require complex approaches for their optimization.^1−4^

New strategies have resulted in several improvements of **zT** throughout the last two decades. Chalcogenide materials dominate the field in the mid-temperature range, based on anharmonic bonding in SnSe, band convergence in PbTe, and defect and disorder engineering in AgSbTe_2_ derivatives.^5−9^ The outstanding performance of AgSbTe_2_ derivatives is also apparent in well-known (GeTe)*_m_*(AgSbTe_2_) TAGS and (PbTe)_*m*_(AgSbTe_2_) LAST derivatives.^10−13^ Nevertheless, in recent years, several articles asserted the high conversion efficiency of AgSbTe_2_ alone. It displays extremely low thermal conductivity, high values of Seebeck coefficient, and a high figure of merit at fairly low temperatures (500–800 K), filling the vacant range left by SnSe and PbTe.^14−16^ For instance, Cao *et al.*(17) obtained a peak **zT** of 1.15 at 623 K, while Wu *et al.*(18) described a peak *zT* of 1.2 at 500 K in Ag_0.9_Sb_1.1_Te_2_, and the Biswas group reported a *zT* as high as 2.4 at 573 K in Yb-doped AgSbTe_2_.^19^ However, there are several inconsistencies concerning its electrical conductivity, affected by crystalline disorder and different nanoprecipitates, mainly Ag_2_Te, which strongly alter the transport properties. Roychowdhury *et al.*(20) described outstanding values of average and peak performance, with *zT* ≈ 2.6 at 573 K, by employing atomic ordering and a systematic approach to improve carrier mobility in this system through Cd doping, which have been then reproduced using Hg as dopant.^21^

AgSbTe_2_ displays a cubic structure in the *Pm*3*m* space group with Ag and Sb sharing 3*c* Wyckoff positions, although most of the literature describes it in more symmetric approximations such as in ref (22). This random Ag/Sb arrangement suppresses the carrier mobility by the disorder-induced localization of electronic states. Besides, several dopants have been studied in this system,^23−26^ resulting in high-performance derivatives with a variety of phenomenologies giving rise to prominent thermoelectric figures of merit (*zT* > 1). The ultralow thermal conductivity and good electrical performance have been also ascribed to the precipitates of Ag_2_Te and Sb_2_Te_3_ formed in nanostructured samples.^27,28^ Nevertheless, the effect of nanostructures in the reduced lattice thermal conductivity has been cast aside by the importance of the s^2^ electron lone pairs in I–V–VI_2_ materials as the main cause of anharmonic bonding.^29^ This pronounced anharmonicity leads to an elevated Grüneisen parameter and strong interactions in phonon–phonon processes.^30^ Theoretical calculations indicate that a significant contrast in force constants arising from the distinct nature of Te–Ag and Te–Sb bonds induces this strong scattering effect on phonons. Consequently, the thermal conductivity of the AgSbTe_2_ compound is constrained by phonon–phonon Umklapp processes.^24^ Additionally, the flat valence band maximum and multipeak valence band structure produce a large positive Seebeck coefficient.^31^

Compositional engineering by the introduction of Ag vacancies leads to the delocalization of electronic states in the system, inducing a heightened separation between the Fermi level and the mobility edge within the electronic structure.^32^ This contributes to a subsequent increase in the power factor and additionally limits the formation of Ag_2_Te impurities in the samples. Consequently, polycrystalline samples of off-stoichiometric Ag_0.7_Sb_1.12_Te_2_ and Ag_0.7_Sb_1.12_Te_1.95_Se_0.05_ were studied in this work. The samples have been synthesized by arc-melting and their properties characterized. The arc-melting synthesis method is very fast, leading to pure phases and dense samples in a straightforward procedure.^33,34^ The crystalline structure of the samples was analyzed by laboratory XRD and synchrotron XRD, showing conspicuous changes in the lattice and anisotropic atomic displacement parameters (ADP) compared to stoichiometric AgSbTe_2_. Moreover, the large weighted mobility and reduced lattice thermal conductivity found in the samples lead to a peak in *zT* of 1.25 for Ag_0.7_Sb_1.12_Te_2_ at 750 K.

## Experimental Methods

Ag_0.7_Sb_1.12_Te_2_ and Ag_0.7_Sb_1.12_Te_1.95_Se_0.05_ have been synthesized by arc-melting from the stoichiometric mixture of the metallic elements Ag (99.97%, Goodfellow Metals, Cambridgeshire, UK), Sb (99.5%, Alfa Aesar, Haverhill, MA, USA), Te (99.99%, Alfa Aesar, Haverhill, MA, USA), and Se (99.5%, Sigma-Aldrich). A 13 mm diameter cold-pressed pellet of the reagents is introduced into the chamber of an Edmund Buhler MAM-1 arc furnace, which is then melted by the electron arc in a water-cooled Cu crucible in an inert Ar atmosphere. In a time span of less than 3 min, the samples are melted three times to ensure homogenization. The resulting ingot is then partially cold-pressed in a disk shape with 8 mm diameter and 3 mm thickness using a Retsch (Haan, Germany) Pellet Press PP25 under an isostatic pressure of 10 MPa to facilitate the transport measurements. Small pieces are cut and ground to powder to perform the structural characterization. The density of the cold-pressed pellet, measured by an Archimedes balance, was >94% of the theoretical crystallographic density.

Preliminary crystallographic characterization was performed for the ground sample using powder X-ray diffraction (XRD) on a Bruker-AXS D8 (Karlsruhe, Germany) diffractometer run by DIFFRACTPLUS software (version 2.5.0, Bruker, Karlsruhe, Germany) with Cu Kα radiation (λ = 1.5418 Å) in Bragg–Brentano reflection geometry.

High-quality synchrotron X-ray diffraction (SXRD) experiments were conducted at the ID22 beamline (European Synchrotron Radiation Facility-ESRF in Grenoble, France).^35^ The incident X-ray radiation had a precise wavelength of 0.35429 Å. The powders of the studied samples were carefully loaded into quartz capillaries with a diameter of 0.7 mm. Diffraction data were collected within the 2θ range of 0.008–47° using a multianalyzer stage equipped with 13 Si(111) crystals. Subsequent Rietveld refinements were performed using the FullProf program.^36^ Peak shapes were characterized using a pseudo-Voigt function, while the refinement process encompassed the following parameters: scale factors, zero-error, background coefficients, asymmetry correction factors, lattice parameters, atomic positions, occupancy factors, and anisotropic displacement parameters.

Differential scanning calorimetry (DSC) analysis was performed in a TA Instruments SDT Q600 instrument with a 20 mg sample from room temperature to 500 °C under an argon flow at a heating rate of 10 K min^–1^ (Supporting Information, section SI1).

Microstructure of the samples was analyzed using scanning electron microscopy (SEM) with an EDX-equipped JEOL 6400 microscope, while high-resolution transmission electron microscopy (HRTEM) and selected-area electron diffraction (SAED) were performed using a JEOL 3000F microscope. Treatment of the HRTEM images was carried out using the Digital Micrograph package.^37^

The thermoelectric characterization of the materials was done using pellets directly obtained after cold pressing the arc-melted products (raw ingots) into 8 mm diameter pellets. To achieve the configuration for specific measurements, a primary cut was made perpendicular to the pressing direction, resulting in two thinner pellets, each approximately 1.2 mm in thickness. On one of these pellets, a further cut was performed parallel to the pressing direction, making a bar-shaped segment dedicated to Seebeck coefficient measurements. Simultaneously, the remaining portion of the pellet was employed for the determination of the electrical resistivity. For thermal diffusivity measurements, a thin slice (approximately 0.3 mm thick) of the original pellet was cut using a diamond saw, polished, and covered with graphite paint. The cut into thinner pellets and bar-shaped pieces was done by using an Isomet low-speed saw (Buehler). A diagram illustrating how the different sections of the sample were cut is given in the Supporting Information, section SI2.

The Seebeck coefficient of the samples was determined by using a commercial MMR-Technologies system. These measurements were conducted under vacuum conditions (10^–3^ mbar) within a temperature range spanning from 300 to 800 K. As a reference, constantan wire was also measured for comparison with the bar-shaped samples, which had been previously cut with a diamond saw perpendicular to the pressing direction. The thermal gradient applied during the Seebeck measurement was oriented perpendicular to the pressing direction. The electrical resistivity measurements were performed using the same instrument, employing the van der Pauw method. For the measurement of thermal diffusivity (α), a Linseis LFA 1000 instrument was employed, and measurements were taken within the temperature range of 300–800 K under an argon atmosphere. To enhance the heat absorption and emissivity, a thin graphite coating was applied to the surface of the pellets. The measurements were taken along the pressing direction. The thermal conductivity (κ) was calculated using the formula κ = α*C*_p_*d*, where *d* stands for the sample density and *C*_p_ represents the specific heat that was estimated using the Dulong–Petit law. κ_L_ is derived by deducting the electronic contribution, κ_e_, from κ, and κ_e_ is determined using the Wiedemann–Franz law: κ_e_ = *L*σ*T* (where *L* represents the Lorenz number). The reported measurements incorporate error margins of 5% for the Seebeck coefficient, 5% for thermal conductivity, and 1.5% for electrical resistivity.

The Hall mobility and carrier density were calculated from the Hall effect and magnetoresistance measured using the four-probe resistivity option of the PPMS (Physical Property Measurement System, by Quantum Design) in the van der Pauw geometry in delta-mode with a DC current of *I* = 5 mA as a function of the magnetic field up to ±8 T at 300 K.

## Results and Discussion

### Laboratory and Synchrotron X-ray Powder Diffraction

**Figure 1:**
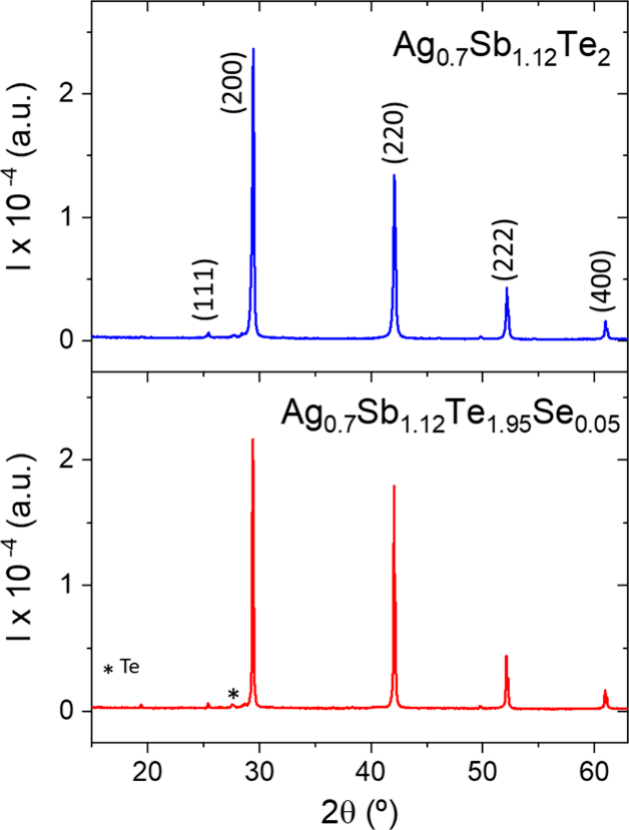
X-ray diffraction patterns of arc-melted Ag_0.7_Sb_1.12_Te_2_ and Ag_0.7_Sb_1.12_Te_1.95_Se_0.05_ (nominal compositions). Miller indices corresponding to cubic *Pm*3*m* reflections are also labeled.

The samples were directly obtained as dense disk-shaped pellets with metallic luster and resistance to fractures. Laboratory XRD characterization (Figure 1) of the ground samples displays the characteristic AgSbTe_2_-type cubic *Pm*3*m* structure. It is noteworthy that the typical Ag_2_Te impurity peaks are not present in either composition studied here, as confirmed by differential scanning calorimetry (DSC) analysis (see Supporting Information, section SI1). In our previous work on arc-melted AgSbTe_2_, the presence of Ag_2_Te was almost negligible in the XRD pattern, but still detectable.^38^ The low amount of impurities has been reported as a consequence of quenching the melted material in the synthesis process, which thwarts the formation of higher melting point impurities.^25^ Furthermore, even those minor amounts of impurities vanish in the samples described here. However, a very small amount of Te (<2%) is observed in the X-ray diffractogram as a secondary phase. Other reports have described the antibonding-induced instabilities in the AgSbTe_2_ electronic structure, which are reduced by Ag vacancies in the crystalline structure. Then, the charge imbalance is compensated by Sb insertion at Ag positions (Sb_Ag_), leading to a pristine structure with low a hole-carrier concentration of ∼1 × 10^18^ cm^–3^. The formation of an Sb-rich matrix is linked to the segregation of Ag_2_Te impurities, which is very common in other AgSbTe_2_ works. Here, by using compositional engineering, the intentionally induced Ag vacancies and Sb excess result in the desired phases. The nominal compositions analyzed here were chosen with slight variations of the stoichiometry upon formation of samples, which follow the stable stoichiometric range described in other reports.^29^

**Figure 2:**
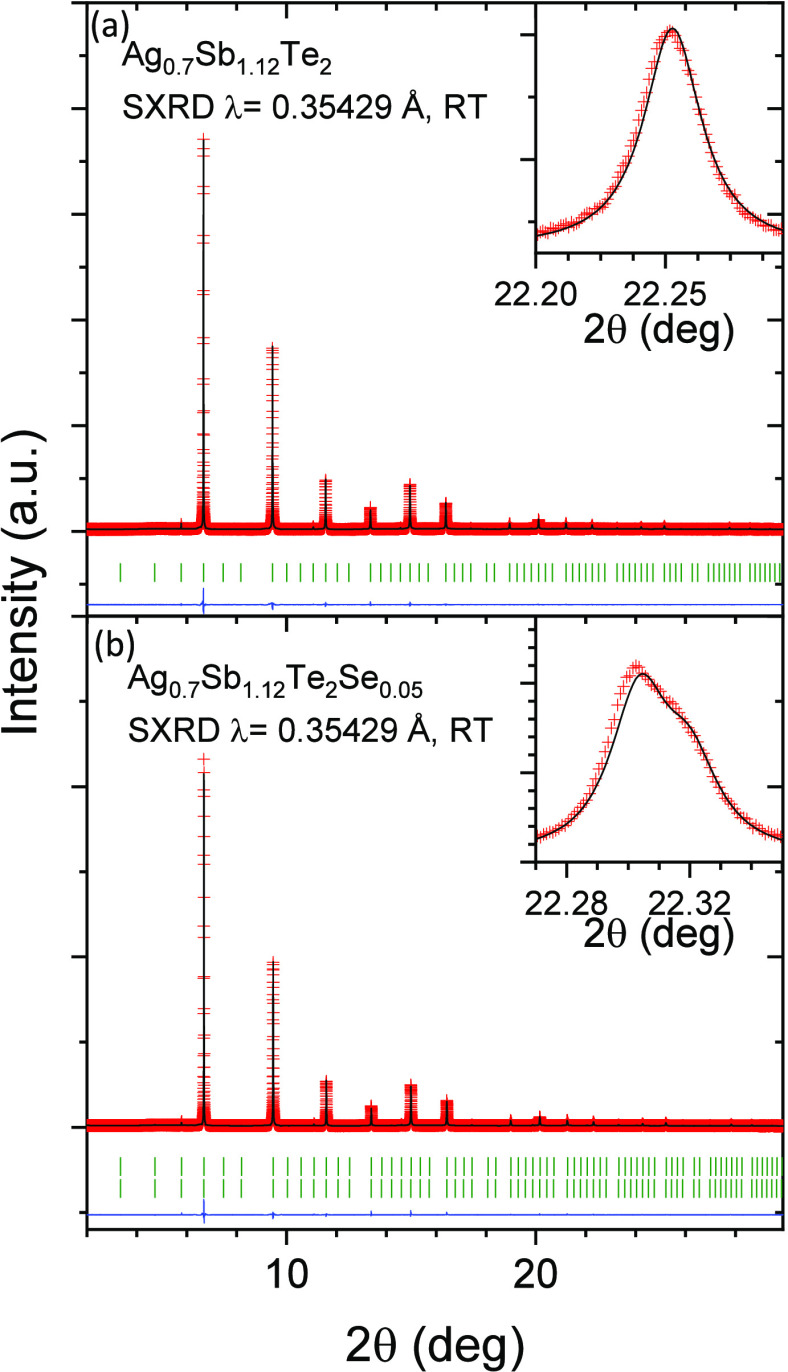
Synchrotron XRD patterns of nominal (a) Ag_0.7_Sb_1.12_Te_2_ and (b) Ag_0.7_Sb_1.12_Te_1.95_Se_0.05_ at room temperature. The experimental data are shown as red crosses, the calculated pattern is shown as a black line, and the difference is shown as a blue line. The insets display (a) a single peak profile and (b) the typical profile for two overlapping peaks. There are no observed secondary phases within the detection limits.

A detailed structural characterization has been performed using high-resolution synchrotron XRD data collected at the ID22 beamline (ESRF) at RT. The higher radiation flux and great resolution of this instrument allow the analysis of the occupation factors and anisotropic displacement parameters (ADPs) by Rietveld refinement of the diffraction patterns. The calculated profiles are defined in the cubic *Pm*3*m* (No. 221) space group and display a very good agreement with experimental data (Figure 2a,b). Using this description, Sb is located at 1*b* (1/2, 1/2, 1/2), Ag and Sb atoms are located at 3*c* (1/2, 1/2, 0), and Te is located at 1*a* (0, 0, 0) and 3*d* (0, 1/2, 0) Wyckoff positions. Structural parameters resulting from the refinement are shown in Table 1. This is a rock-salt-like crystallographic structure with every position showing an octahedral configuration and equal average bond lengths. In nominal Ag_0.7_Sb_1.12_Te_2_, the Ag deficiency and Sb excess are determined in the refinement, showing values similar to those from EDX analysis (see Supporting Information, section SI3).

**Table 1:** Structural Parameters Calculated from the Rietveld Refinement of Nominal Compositions Ag_0.7_Sb_1.12_Te_2_ and Ag_0.7_Sb_1.12_Te_1.95_Se_0.05_ SXRD Data at Room Temperature

nominal composition	Ag_0.7_Sb_1.12_Te_2_	refined composition	Ag_0.86(4)_Sb_1.06(4)_Te_1.93(1)_
lattice parameter (Å)	6.08918(2)	cell volume (Å^3^)	225.7748(9)

atomic displacement parameters (Å^2^)				
atom	*U*^11^	*U*^22^	*U*^33^	occ (<1)
Te1	0.0321(7)	0.031(1)	0.0321(7)	1.000
Te2	0.039(2)	0.039(2)	0.039(2)	0.850(5)
Sb	0.030(2)	0.030(2)	0.030(2)	1.000
Sb2	0.0352(9)	0.0352(9)	0.072 (2)	0.37(2)
Ag	0.0352(9)	0.0352(9)	0.072 (2)	0.58(2)

agreement factors					bonding distance (Å)
*R*_I_ (%)	*R*_p_ (%)	*R*_wp_ (%)	*R*_exp_ (%)	χ^2^	*d*(Ag/Sb–Te)
2.3	2.3	3.0	1.4	4.7	3.04459(2)

nominal composition Ag_0.7_Sb_1.12_Te_1.95_Se_0.05_		
refined composition	Ag_0.8_Sb_1.14_Te_1.91_Se_0.09_	Ag_0.8_Sb_1.14_Te_1.97_Se_0.03_
phase abundance (%)	33.9(2)	66.1(2)
lattice parameter (Å)	6.07103(2)	6.07544(2)
cell volume (Å^3^)	223.762(1)	224.251(1)

atomic displacement parameters (Å^2^)					
				occ (<1)	
atom	*U*^11^	*U*^22^	*U*^33^		
Te1	0.0282(4)	0.0295(9)	0.0282(4)	1.00	1.00
Te2	0.051(1)	0.051(1)	0.051(1)	0.82	0.94
Se	0.051(1)	0.051(1)	0.051(1)	0.18	0.06
Sb	0.0246(9)	0.0246(9)	0.0246(9)	1.00	1.00
Sb2	0.0308(5)	0.0308(5)	0.063(2)	0.43	0.43
Ag	0.0308(5)	0.0308(5)	0.063(2)	0.53	0.53

agreement factors						bonding distance (Å)	
*R*_I_ (%)						*d*(Ag/Sb–Te)	
		*R*_p_ (%)	*R*_wp_ (%)	*R*_exp_ (%)	χ^2^		
2.63	2.60	2.3	3.1	1.5	4.2	3.03551(2)	3.03772(2)

**Figure 3:**
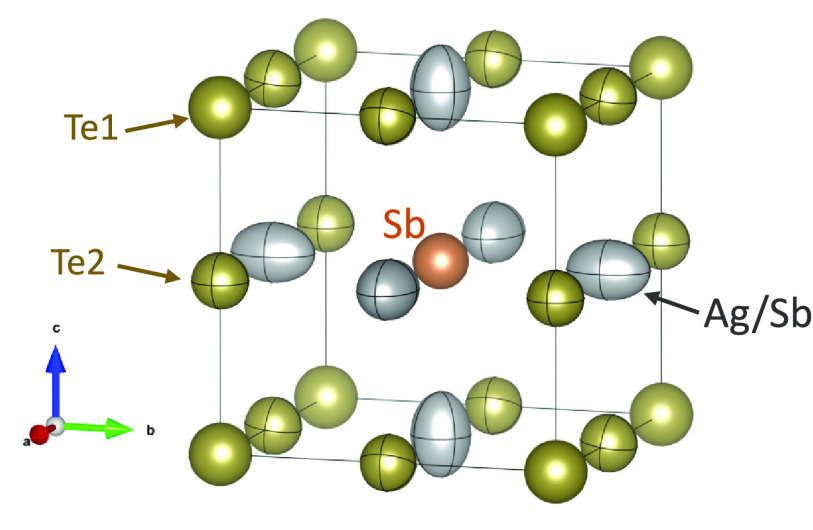
Representation of refined Ag_0.7_Sb_1.12_Te_2_ crystal structure, including the ADP as ellipsoids determined by crystal symmetry.

Despite the induced vacancies in the crystal structure and the reduced covalent radius of Sb (1.39 Å) vs Ag (1.45 Å),^39^ the lattice parameter of nominal Ag_0.7_Sb_1.12_Te_2_ increases with respect to stoichiometric AgSbTe_2_ (6.0788 Å).^38^ This expansion of the unit cell agrees with a reduced bonding strength, with bond length increasing from *d*(Ag/Sb–Te) = 3.03939 Å to *d*(Ag/Sb–Te) = 3.04459(4) Å, which can be a consequence of increased Sb_Ag_ substitution and filling of antibonding states as previously described.^40^ The reduced bond order can lead to reduced lattice thermal conductivity, altered bandwidth, and effective mass. The representation of the Ag_0.7_Sb_1.12_Te_2_ structure is shown in Figure 3. In our previous report on stoichiometric AgSbTe_2_, the ADP ellipsoids of Ag/Sb atoms at 3*c* positions displayed a disk-like shape elongated along Ag/Sb–Te bonds (Supporting Information, section SI4). However, here, the ADP ellipsoids are hugely elongated along Ag/Sb–Sb bonds, indicating weakened interactions. Comparing absolute values, the 3*c* ADP values are still similar to those of the stoichiometric composition in the *bc* plane (along Ag/Sb–Te bonds), while there is an overall increase in every position. Furthermore, the ellipsoids at Te1 show much larger *U*^11^ and *U*^33^ and slightly reduced *U*^22^, suggesting more homogeneous surroundings, which would be in agreement with weaker Sb/Ag–Te interactions. This scheme agrees well with weakened bond order in the structure, in which there is a significant increase of static and dynamic disorder at the 3*c*(Sb/Ag) position.

The diffraction pattern of Ag_0.7_Sb_1.12_Te_1.95_Se_0.05_ displays a conspicuous splitting of the peaks, more visible at high diffraction angles, as shown in the inset of Figure 2b. This indicates a phase segregation into two cubic phases with a slight variation of the lattice parameter and thus either different structural order or phase composition. The segregation into domains with different composition has been previously described by microscopic analysis and is observed here thanks to the high resolution of the synchrotron experiment.^41^ This segregation is proposed as two differently Se-doped phases, of which the occupation factors were fixed based on elemental analysis by EDX. The fast solidification of melted particles in complex systems can lead to localized temperature gradients and might result in layered cooling, producing small variations in the composition of the segregated phases.^42^ The best agreement factors were obtained by placing Se atoms at the 1*a* Wyckoff positions using the occupation factors described in Table 1. Any attempts to introduce Se at 3*c* Wyckoff positions were unsuccessful, leading to unrealistic compositions. This suggests that the substitution of Te2 by Se is the most favorable configuration. As a consequence of the smaller covalent radius of Se (1.20 Å) compared to Te (1.38 Å),^39^ there is a reduction of the lattice parameter to values similar to other Se-doped samples in the literature.^25^ The ADP values of this sample show an arrangement similar to that in the undoped off-stoichiometric composition, as the ellipsoids at 3*c* positions are elongated along Ag/Sb–Sb bonds. Additionally, the Te1/Se position shows larger disorder with increased ADP values, as expected for the shared crystallographic position. Overall, the Ag- and Sb-off stoichiometry and Se doping induces further weakening of bonding interactions.

### SEM and HRTEM Microscopy

**Figure 4:**
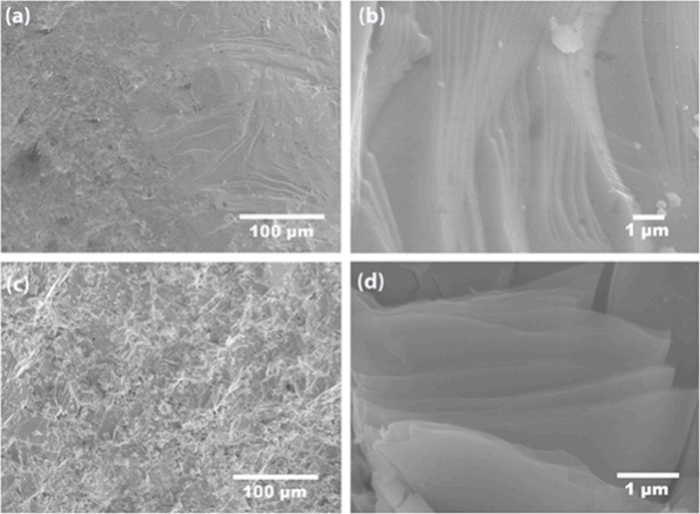
SEM micrographs of arc-melted (a, b) Ag_0.7_Sb_1.12_Te_2_ and (c, d) Ag_0.7_Sb_1.12_Te_1.95_Se_0.05_.

SEM micrographs (Figure 4) show a compact morphology in the pellets, with the typical layered structure found for arc-melted samples.^33,38,43^Figure 4a,b shows the microstructure of Ag_0.7_Sb_1.12_Te_2_, while the morphology of Ag_0.7_Sb_1.12_Te_1.95_Se_0.05_ is depicted in Figure 4c,d. Figure 4a,c shows high densification over large areas of the compacted pellets (*ca.* 450 μm), whereas micrographs of the cross section of the pellets are included in Figure 4b,d. The melting in an arc furnace results in strongly textured materials, consisting of stacked sheets, on a nanoscale in one direction. The nanostructuring strongly influences the thermoelectric properties of these materials, providing surface boundaries that induce a significant scattering of phonons and electrons.^44^ The actual composition, assessed semiquantitatively using EDX in over ten individual crystals, shows a small deviation from the nominal composition, as Ag_0.9_Sb_1.04_Te_2_ and Ag_0.79_Sb_1.13_Te_1.87_Se_0.06_ (Supporting Information, section SI3), which agrees with the best Rietveld refinements of the synchrotron X-ray diffraction data.

**Figure 5:**
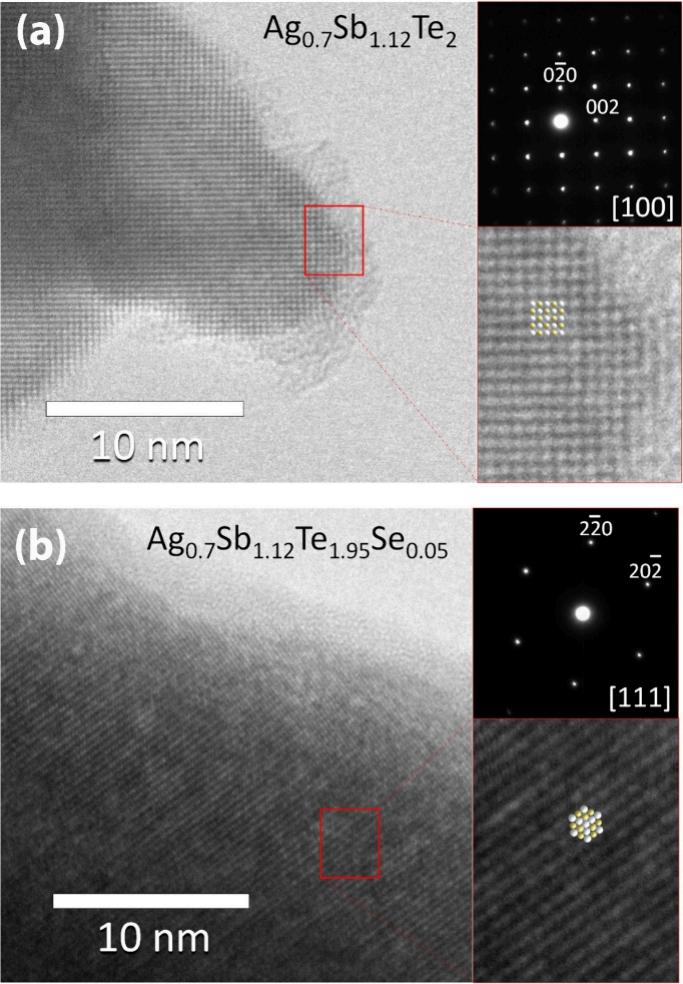
Experimental HRTEM micrographs of nominal (a) Ag_0.7_Sb_1.12_Te_2_ along the [100] zone axis and (b) Ag_0.7_Sb_1.12_Te_1.95_Se_0.05_ along the [111] zone axis. The upper insets on the right of both panels display SAED patterns, while the bottom insets offer a magnified view of a specific region within the HRTEM images, as indicated by the red rectangle.

The crystals of the two arc-melted samples were examined using high-resolution transmission electron microscopy (HRTEM) and selected area electron diffraction (SAED). Figure 5a shows the experimental image captured along the [100] zone axis of the Ag_0.7_Sb_1.12_Te_2_ sample, while Figure 5b depicts the Ag_0.7_Sb_1.12_Te_1.95_Se_0.05_ HRTEM micrograph along the [111] zone axis. In both images, the dots with color contrast represent the atomic columns’ projections stemming from the Ag/Sb and Te/Se sublattices, revealing a highly crystalline and ordered structure. The study of the reciprocal space by SAED, shown in the upper panel of both micrographs, confirms the cubic structure with a cell parameter of ca. 6.07 Å, in good agreement with the information extracted from synchrotron X-ray diffraction. The lack of additional reflections, streaking lines, or diffuse scattering among the main reflections confirms the absence of extended defects, superstructures, dislocations, etc. Enlargements of the HRTEM images, with the corresponding model projections (Ag/Sb in gray and Te/Se in yellow) overlaid on the experimental images for each material, are presented in the bottom part of both figure panels, demonstrating a strong level of agreement between the model and the micrographs.

### Thermoelectric Properties

**Figure 6:**
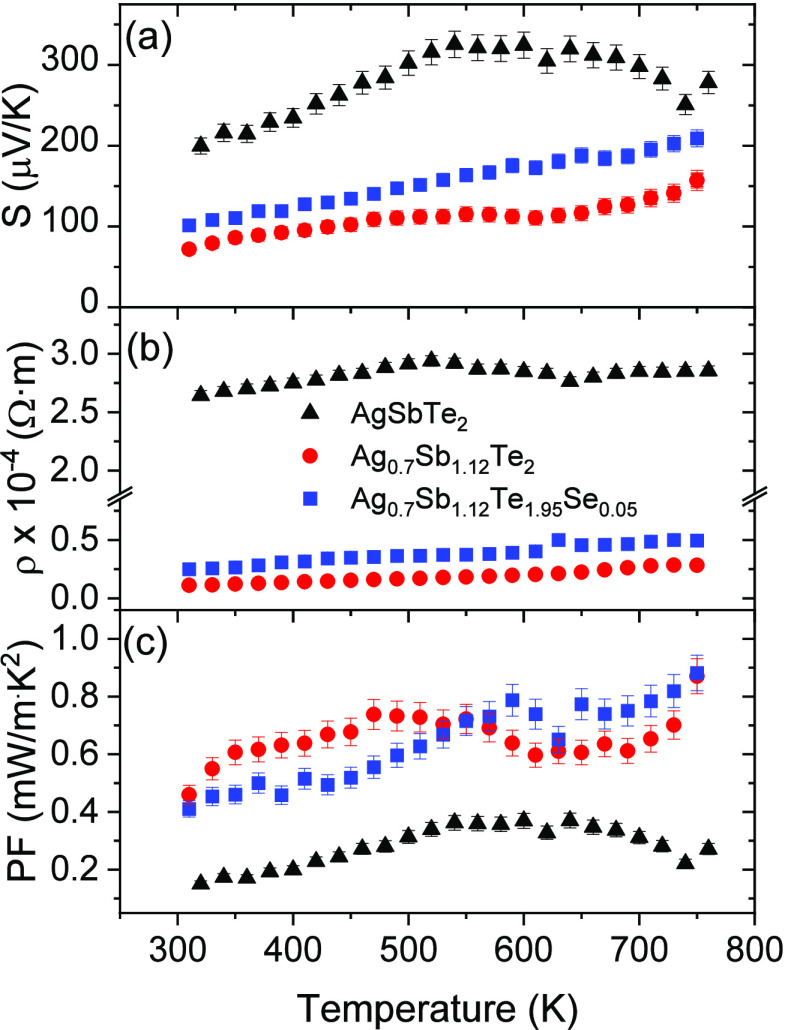
Temperature dependence of (a) the Seebeck coefficient (*S*), (b) electrical resistivity (ρ), and (c) power factor (PF = *S*^2^/ρ) of the arc-melted AgSbTe_2_-based samples. Error bars are indicated in the graphics.

The evolution of the electrical transport properties of Ag_0.7_Sb_1.12_Te_2_ and Ag_0.7_Sb_1.12_Te_1.95_Se_0.05_ with temperature is shown in Figure 6 and compared with previously reported^38^ similarly synthesized stoichiometric AgSbTe_2_. A monotonic increase in the Seebeck coefficient is observed as the temperature increases (Figure 6a) in both Ag-deficient samples. However, a maximum Seebeck value is not reached within the measurement range of the equipment and, considering that the melting point is around 830 K, there is no room for measurement at higher temperatures. All samples exhibit a positive Seebeck coefficient, indicating that the dominant charge carriers for the prepared compositions are holes (p-type conductivity). Although in AgSbTe_2_ a bipolar contribution is evident as there is initially an increment of the Seebeck coefficient, followed by a plateau up to 700 K followed by a sharp reduction, this behavior is not visible in the measured temperature range for the nonstoichiometric samples in this study. Maximum values of the Seebeck coefficients of 157 and 209 μV K^–1^ were measured for Ag_0.7_Sb_1.12_Te_2_ and Ag_0.7_Sb_1.12_Te_1.95_Se_0.05_, respectively, both at 750 K. These values are notably lower than the Seebeck coefficient of 340 V K^–1^ at 540 K reported for the arc-melted AgSbTe_2_ reference.^38^ This decrease might be associated with an increase in the carrier concentration, which is consistent with the significant variation in electrical resistivity, reduced by 1 order of magnitude through compositional engineering in Ag-deficient samples (Figure 6b). The electrical properties of pristine AgSbTe_2_ are mainly hindered by its high electrical resistivity because of deficient carrier concentration,^8^ while using this vacancy-engineering strategy might produce an enhancement of the number of charge carriers, similar to what has been reported for other aliovalent doped samples.^15,23,26,45,46^ Furthermore, the synthesis method also plays a critical role in the electrical properties. As an example, it has been reported that ball-milling AgSbTe_2_ induces changes of carrier density (due to the presence of defects), and in this case, nanostructuration produced by arc-melting also affects the electrical properties. In this work, the resistivity of the Ag-deficient samples (Figure 6b) increases slightly with temperature, from 1.2 × 10^–5^ to 2.9 × 10^–5^ Ω m between 300 and 750 K for Ag_0.7_Sb_1.12_Te_2_ and 2.3 × 10^–5^ to 5.1 × 10^–5^ Ω m at the same temperature range for Ag_0.7_Sb_1.12_Te_1.95_Se_0.05._

As a result, a power factor of close to 0.9 mW m^–1^ K^–2^ at 750 K is calculated for both samples (Figure 6c). This power factor value is approximately 125% higher than the maximum value found for the AgSbTe_2_ arc-melted sample (0.4 mW m^–1^ K^–2^ at 630 K). However, it is worth noting that more recently, higher power factors, up to 2.1 mW m^–1^ K^–2^ at 673 K, have been identified in AgSbTe_1.85_Se_0.1_S_0.05_ system, where the charge carrier density and phase stability of polycrystalline AgSbTe_2_ were optimized by doping with S and Se.^40^

**Figure 7:**
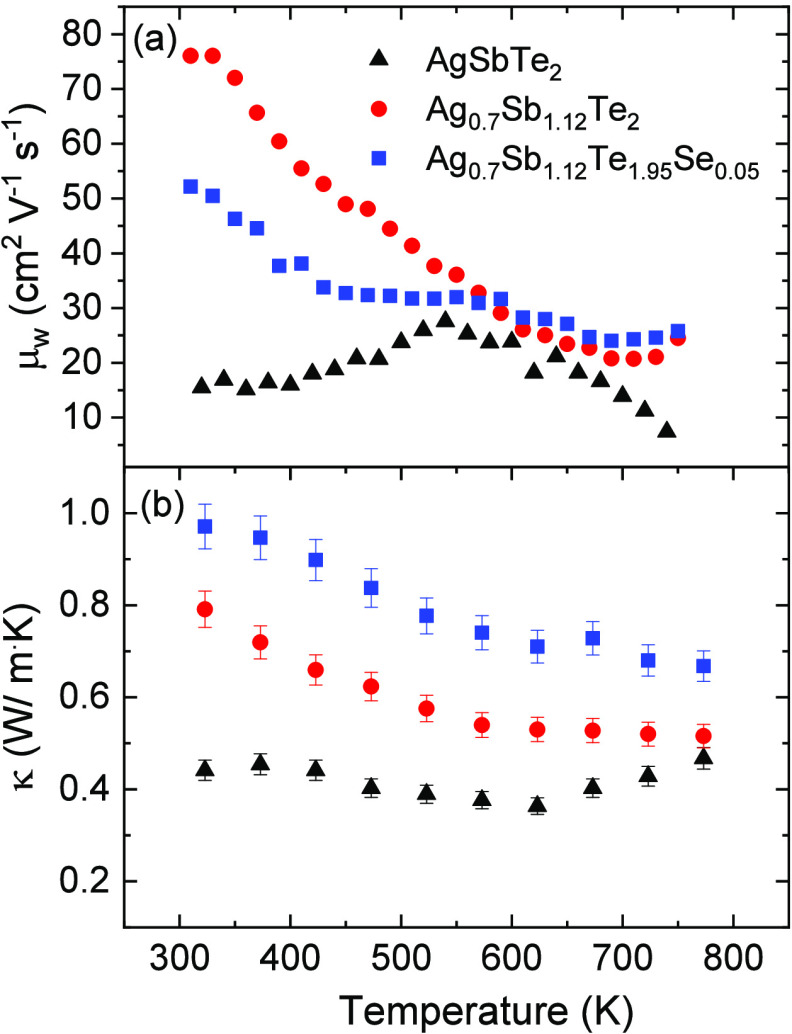
Temperature dependence of (a) weighted mobility and (b) total thermal conductivity of the arc-melted AgSbTe_2_-based samples. Error bars are indicated in the graphics.

Figure 7a represents the temperature dependence of the weighted mobility of the arc-melted samples. Characterizing charge carrier mobility through experimental means is of paramount importance for comprehending and engineering thermoelectric materials. This parameter can easily be calculated from the measured values of the Seebeck coefficient and the electrical conductivity as described by Snyder *et al.*(47) As expected, this parameter is higher for Ag_0.7_Sb_1.12_Te_2_ and Ag_0.7_Sb_1.12_Te_1.95_Se_0.05_ than for AgSbTe_2_, due to the higher purity of the samples, as lattice boundaries with Ag_2_Te impurities and different Sb-rich domains are not present in these samples by using phase-pure compositions. Moreover, Ag-deficient samples have been shown to increase cationic Ag/Sb order and allow for better carrier mobility in this material, as Ag vacancies favor the rearrangement of cation in the AgSbTe_2_ structure.^32^ At room temperature, the maximum value of weighted mobility was 76 cm^2^ V^–1^ s^–1^ in Ag_0.7_Sb_1.12_Te_2_, whereas stoichiometric AgSbTe_2_ exhibits a value of 15 cm^2^ V^–1^ s^–1^. This disparity becomes less pronounced at higher temperatures, with values of 7, 24, and 26 cm^2^ V^–1^ s^–1^ for AgSbTe_2_, Ag_0.7_Sb_1.12_Te_2_, and Ag_0.7_Sb_1.12_Te_1.95_Se_0.05_, respectively. The weighted mobility agrees well with the Hall mobility of the minority n-type carriers at 300 K but is an order of magnitude higher than that of the majority carriers (Hall mobility and carrier concentrations are given in Supporting Information, section SI5), calculated using the two-band model of Jovovic *et al.*(48))

**Figure 8:**
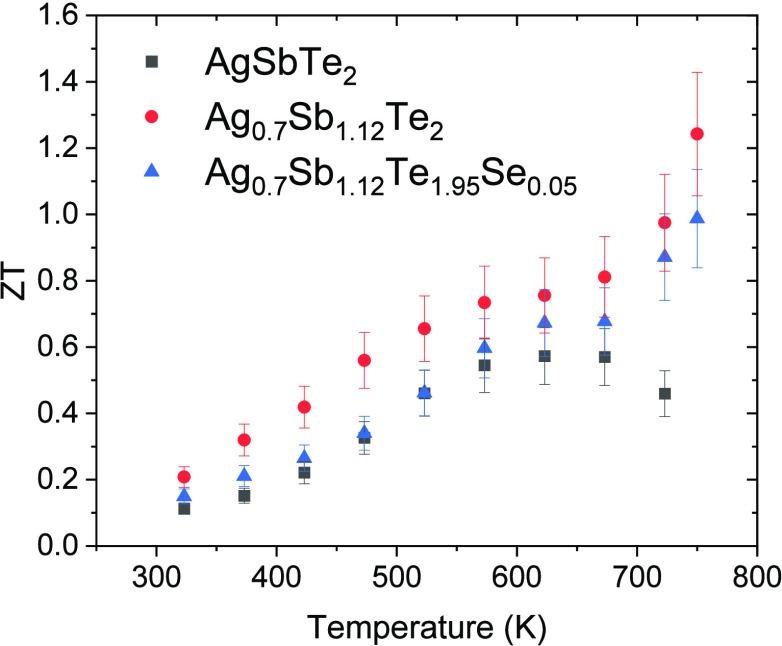
Temperature dependence of the figure of merit (*zT*) of the arc-melted AgSbTe_2_-based samples. Error bars are indicated in the graphic.

AgSbTe_2_ presents an exceptionally low intrinsic thermal conductivity due to the anharmonic nature of the Sb–Te bonds, which is primarily attributed to the stereochemically active 5s^2^ lone pairs of Sb atoms. This anharmonicity is a consequence of the electrostatic repulsion between the lone electron pairs of Sb and the valence bonding charge of Se. Furthermore, the disorder of Ag/Sb on the face-centered lattice reduces the thermal conductivity, as it creates effective scattering of heat-carrying phonons. In the Ag-deficient samples, synthesized in this work (Ag_0.7_Sb_1.12_Te_2_ and Ag_0.7_Sb_1.12_Te_1.95_Se_0.05_), there is a clear decrease in the thermal conductivity as temperature increases (Figure 7b), although the thermal conductivity is slightly higher at room temperature. This reduction in thermal conductivity eventually leads to values of 0.50 and 0.64 W m^–1^ K^–1^ at 773 K in Ag_0.7_Sb_1.12_Te_2_ and Ag_0.7_Sb_1.12_Te_1.95_Se_0.05_, respectively, which are close to the thermal conductivity value of 0.50 W m^–1^ K^–1^ in AgSbTe_2_. According to the literature, the glass-like thermal conductivity values for AgSbTe_2_ systems fall within a range of 0.4–0.6 W m^–1^ K^–1^,^8,49^ exhibiting a relatively consistent behavior over a range of temperature changes. These low values found in the Ag-deficient samples prepared by arc-melting with the higher electronic conductivity might be attributed to the nanostructured nature of the samples, leading to strong phonon scattering at grain boundaries. The presence of vacancies in an Ag/Sb position and the cation disorder in the rock-salt structure hinder phonon transport due to random fluctuations in the local force constants of Ag/Sb cations. Since Ag and Sb atoms share identical crystallographic positions with varying valence electron configurations, nanoscale deviations from crystalline lattice periodicity emerge in regions enriched with Ag or Sb, resulting in an increased number of phonon scattering centers.^8^ The electronic and lattice contributions of thermal conductivity, along with the verification of reproducibility of the thermoelectric properties across different samples, are detailed in the Supporting Information, sections SI6 and SI7. It is observed that due to the lower electrical conductivity (and reduced total thermal conductivity) in Ag_0.7_Sb_1.12_Te_2_, the lattice thermal conductivity is considerably lower than in Ag_0.7_Sb_1.12_Te_1.95_Se_0.05._ Disordered, al-scale hierarchical morphology has previously shown lattice contribution below the amorphous limit in SnTe, reaching almost negligible values.^4,50^ In AgSbTe_2_, this limit is closer to 0.2 W m^–1^ K^–1^ that our sample slightly crosses at higher temperatures (Supporting Information, section SI6).^19^ Taking into account the enhanced power factor and similar thermal conductivity, the figure of merit (*zT*) calculated for the three samples is higher for Ag_0.7_Sb_1.12_Te_2_ and Ag_0.7_Sb_1.12_Te_1.95_Se_0.05_. In the case of Ag_0.7_Sb_1.12_Te_2,_ a maximum *zT* of 1.25 at 750 K was determined, while Ag_0.7_Sb_1.12_Te_1.95_Se_0.05_ exhibited a maximum of *zT* of 1.01 at the same temperature (Figure 8). These results demonstrate that compositional engineering, achieved by introducing Ag vacancies into the AgSbTe_2_ system, yields properties promising for the thermoelectric energy conversion sector, particularly in the mid-temperature region.

## Conclusions

This study describes the arc-melting synthesis, structural analysis, and thermoelectric characteristics of Ag_0.7_Sb_1.12_Te_2_ and Ag_0.7_Sb_1.12_Te_1.95_Se_0.05_.These Ag-deficient materials were prepared as an approach to understand the effect of compositional engineering by introducing Ag vacancies within the well-known AgSbTe_2_ thermoelectric material. These vacancies induce an enlargement of the unit cell, and this suggests the filling of antibonding states with Sb_Ag_ and the weakening of covalent bonding interactions within the crystalline structure. Additionally, enlargement of ADP ellipsoids indicates further anharmonic bonding and contributes to a reduced lattice thermal conductivity. The nanostructured nature of the samples significantly contributes to their thermoelectric performance, creating effective phonon scattering at grain boundaries. This effect coupled with the anharmonic nature of their chemical bonds generates a glass-like low thermal conductivity, reaching values of 0.50 and 0.64 W m^–1^ K^–1^ at 773 K in Ag_0.7_Sb_1.12_Te_2_ and Ag_0.7_Sb_1.12_Te_1.95_Se_0.05_, respectively. The relatively high Seebeck coefficient values found for these samples, combined with a decrease of an order of magnitude in the resistivity of the Ag-deficient samples compared to arc-melted AgSbTe_2_, result in a power factor of approximately 0.9 mW m^–1^ K^–2^ at 750 K. Furthermore, cation ordering and weakened localization could be related to the reduced scattering of charge carriers, primarily resulting from the stabilization of the AgSbTe_2_ rock-salt structure, particularly by silver vacancies, as observed from the weighted mobility. Consequently, high *zT* figures of merit of 1.25 at 750 K for Ag_0.7_Sb_1.12_Te_2_ and 1.01 for Ag_0.7_Sb_1.12_Te_1.95_Se_0.05_ have been reached. These findings demonstrate that compositional engineering can enhance the thermoelectric properties of materials and offer valuable insights into the development of efficient thermoelectric materials, particularly in the mid-temperature range.

## Supporting Information Available

Differential scanning calorimetry (DSC), sample cutting process, EDX analysis, atomic displacement parameters of nominal AgSbTe_2_, Hall carrier concentration and mobility, thermal conductivity, and reproducibility study (PDF)
