# Supplementary material for: Optimizing Thermoelectric Properties through Compositional Engineering in Ag-Deficient AgSbTe2 Synthesized by Arc Melting
Source: ACS Appl Electron Mater. 2024 Mar 5;6(5):2969–77. doi: 10.1021/acsaelm.3c01653 (PMC11138145; doi:10.1021/acsaelm.3c01653)
Supplement: Supplementary file 1 — el3c01653_si_001.pdf [file el3c01653_si_001.pdf]

Supporting Information

Optimizing thermoelectric properties through compositional engineering in Ag-deficient $AgSbTe_2$ synthesized by arc melting

Jesús Prado-Gonjal[1]*, Elena García-Calvo[1,2], Javier Gainza[2], Oscar J. Durá[3], Catherine Dejoie[4], Norbert M. Nemes[5], José Luis Martínez[2], José Antonio Alonso[2], Federico Serrano-Sánchez[2]*

[1] Departamento de Química Inorgánica, Universidad Complutense de Madrid, Ciudad Universitaria s/n, Madrid, E-28040, Spain.

[2] Instituto de Ciencia de Materiales de Madrid, CSIC, Cantoblanco, Madrid, E-28049, Spain.

[3] Departamento de Física Aplicada, Universidad de Castilla-La Mancha, Ciudad Real, E-13071 Spain.

[4] European Synchrotron Radiation Facility (ESRF), 71 Avenue des Martyrs, 38000 Grenoble, France.

[5] GFMC, Departamento de Física de Materiales, Universidad Complutense de Madrid, Madrid, E-28040 Spain.

*corresponding authors: jpradogo@ucm.es; fserrano@icmm.csic.es

SI1. Differential Scanning Calorimetry (DSC)

SI2. Sample cutting process

SI3. EDX analysis

SI4. Atomic displacement parameters of nominal $AgSbTe_2$

SI5. Hall carrier concentration and mobility

SI6. Thermal conductivity

SI7. Reproducibility study

SI1. Differential Scanning Calorimetry (DSC)

Figure S1 shows the Differential scanning calorimetry (DSC) curves for the $Ag_{0.7}Sb_{1.12}Te_2$ and $Ag_{0.7}Sb_{1.12}Te_{1.95}Se_{0.05}$.

A signal around 418 K has been reported in Ag_2Te, associated with the monoclinic to cubic phase transition, that it is not present in our samples (Cao, J et al (2023). Nano Energy, 107, 108118). DSC results confirms that there is no Ag_2Te impurity in the arc-melting samples, as it was observed by synchrotron X-ray diffraction).

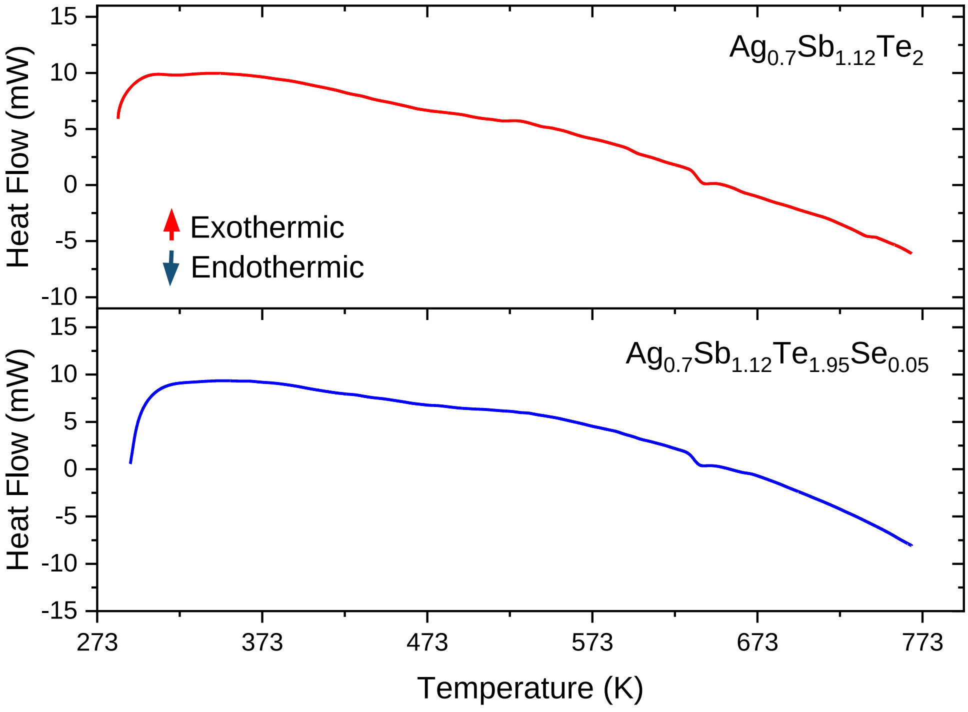

Figure S1. Differential scanning calorimetry (DSC) curves for the $Ag_{0.7}Sb_{1.12}Te_2$ and $Ag_{0.7}Sb_{1.12}Te_{1.95}Se_{0.05}$.

SI2. Sample cutting process

(a)

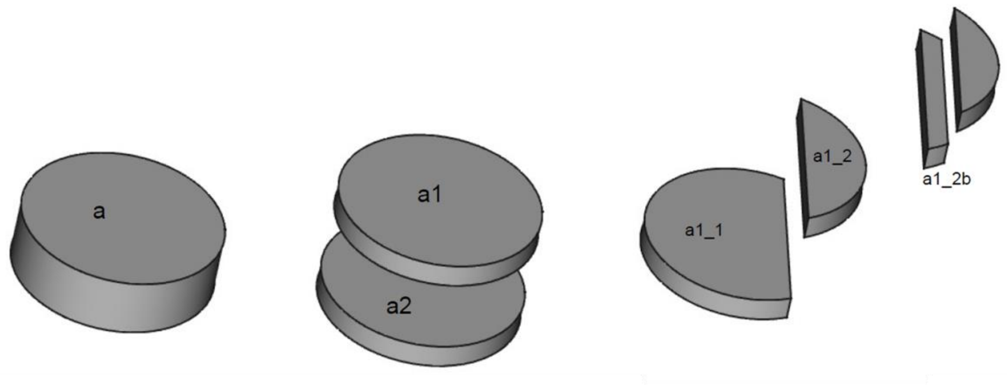

Original sintered pellet (a)

(a) is cut along its section (cut along the in-plane direction). Thus, two similar discs are obtained (a1 and a2). Thermal diffusivity is measured in a2.

One cut perpendicular to the in-plane direction in a1 yields two symmetrical pieces, denoted as a1_1 and a1_2. The resistivity (by van der Pauw method) is measured in a1_1, while a1_2 is further cut into a bar, labeled as a1_2b (and another unused piece). The Seebeck coefficient is measured in a1_2b.

(b)

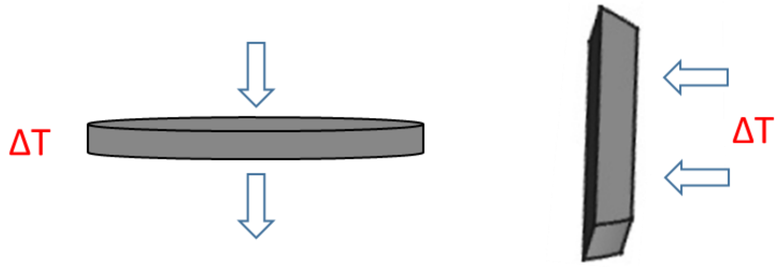

Figure S2. (a) Diagram illustrating how the different sections of the sample were cut for thermoelectric measurements. (b) ΔT and current direction in thermal diffusivity (pellet) and Seebeck coefficient (bar) measurements.

SI3. EDX analyisis

The composition has been assessed semi-quantitatively using Energy Dispersive X-ray Spectroscopy (EDX) on more than ten individual crystals for each sample. Here, we show the average % atomic values in order to compare with the formula achieved by Rietveld refinement of the synchrotron X-ray diffraction data.

1) Rietveld refinement formula: $\mathbf{Ag_{0.86(4)}Sb_{1.06(4)}Te_{1.93(1)}}$

EDX analysis: $\mathbf{Ag_{0.9}Sb_{1.04}Te_{2}}$

Table S1 EDX results in nominal $Ag_{0.7}Sb_{1.12}Te_2$:

Element	% atomic
Ag	20.27
Sb	26.44
Te	53.29

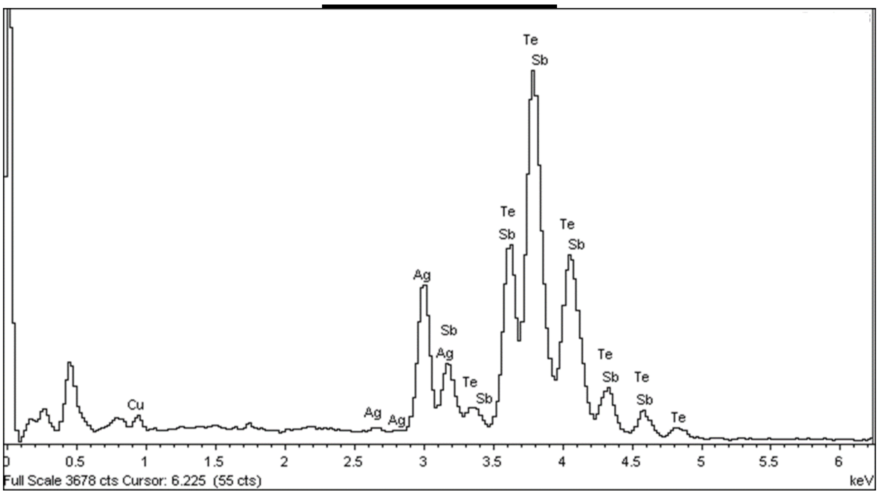

Figure S3. EDX spectra of nominal $Ag_{0.7}Sb_{1.12}Te_2$

2) Rietveld refinement analysis: $\mathbf{Ag_{0.8}Sb_{1.14}Te_{1.91}Se_{0.09}}$ + $\mathbf{Ag_{0.8}Sb_{1.14}Te_{1.97}Se_{0.03}}$ (phase segregation)

EDX analysis: $\mathbf{Ag_{0.79}Sb_{1.13}Te_{1.87}Se_{0.06}}$

Table S2. EDX results in nominal $Ag_{0.7}Sb_{1.12}Te_{1.95}Se_{0.05}$

Element	% atomic
Ag	18.28
Sb	29.52
Te	51.19
Se	1.01

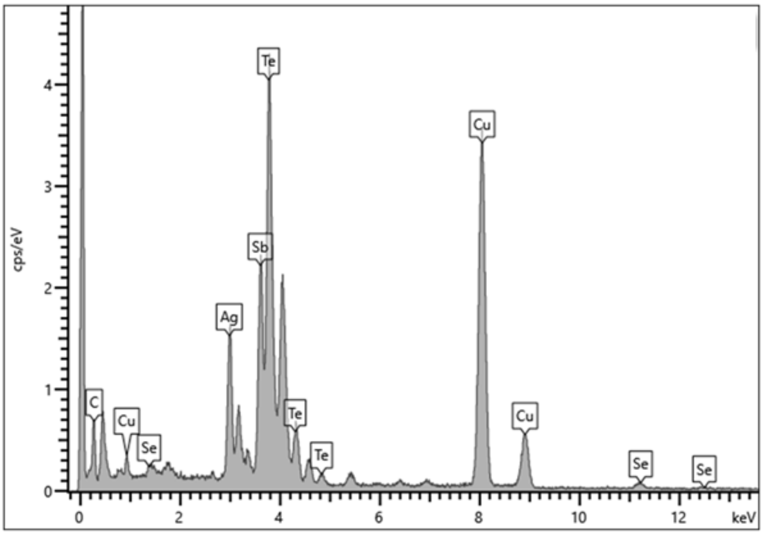

Figure S4. EDX spectra of nominal $Ag_{0.7}Sb_{1.12}Te_{1.95}Se_{0.05}$

SI4. Atomic displacement parameters of nominal $AgSbTe_2$

Table S3. Result of Atomic displacement parameters (ADP) of nominal $AgSbTe_2$, adapted from ref [J. Gainza et al. *Nanomaterials* **2022,** *12* (21), 3910].

	Atomic Displacement Parameters (Å^2)				
	U^{11}	***U^{22}***	***U^{33}***	***U^{12}***	***U^{13}***
Te1	0.022 (5)	0.04 (1)	0.022 (5)	0.00000	0.00000
Sb	0.038 (8)	0.038 (8)	0.038 (8)	0.00000	0.00000
Ag	0.036 (5)	0.036 (5)	0.005 (6)	0.00000	0.00000
Sb2	0.036 (5)	0.036 (5)	0.005 (6)	0.00000	0.00000

SI5. Hall carrier concentration and mobility

Table S4. Hall carrier concentration and mobility at T=300 K of the arc-melted samples.

Nominal Composition	n_H (cm^{-3})	μ_n (cm^2 V^{-1} s^{-1})	p_H (cm^{-3})	μ_p (cm^2 V^{-1} s^{-1})
$Ag_{0.7}Sb_{1.12}Te_2$	$3.7x10^{17}$	64	$1.5x10^{20}$	2.5
$Ag_{0.7}Sb_{1.12}Te_{1.95}Se_{0.05}$	$1.5x10^{16}$	83	$5.7x10^{19}$	1.4

SI6. Thermal conductivity

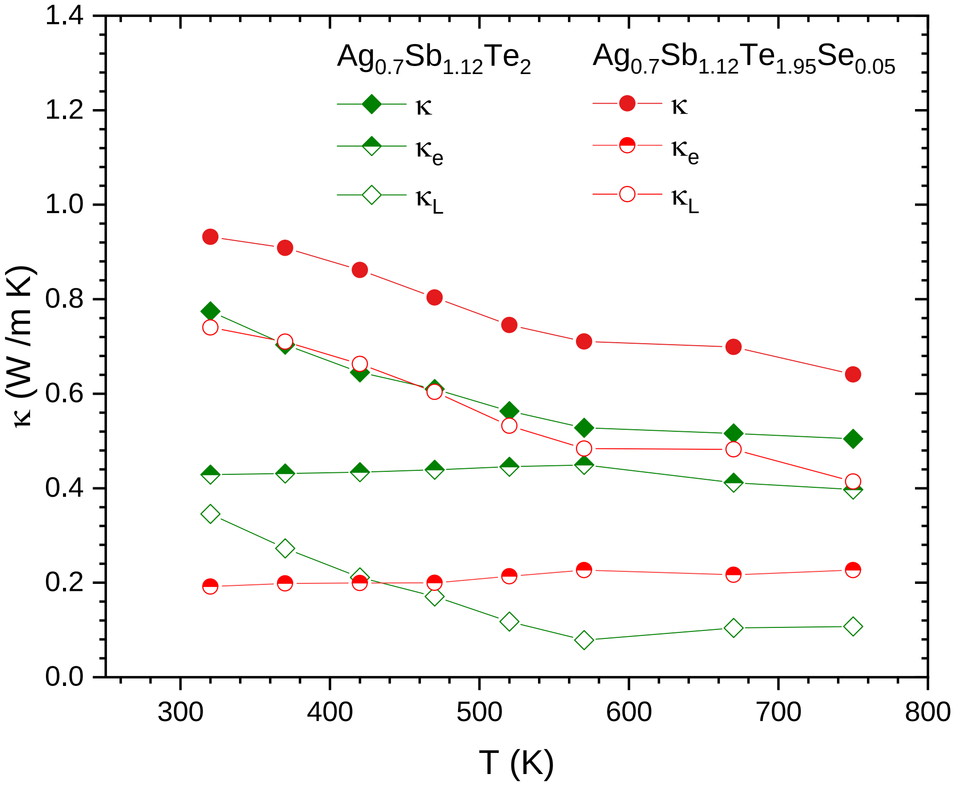

Figure S5 Temperature dependence of the total thermal conductivity and the thermal and electronic contributions of the thermal conductivity.

SI7. Reproducibility study

Two samples have been measured in order to assure the thermoelectric properties of the system.

Figures S6, S7 and S8 shows the temperature dependence of the Seebeck coefficient, electrical conductivity and thermal conductivity.

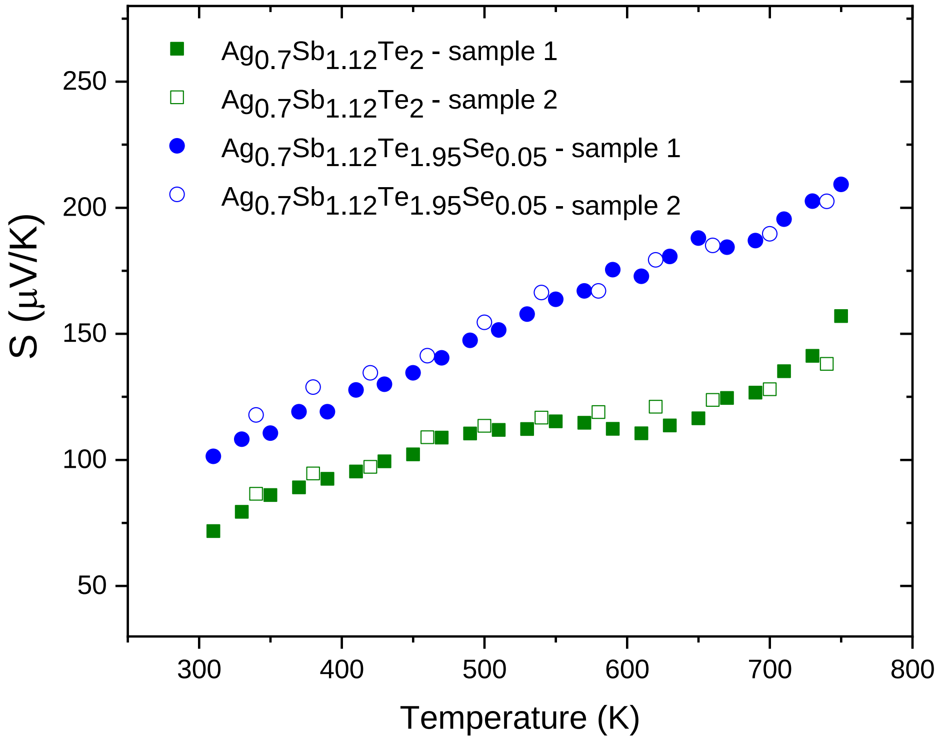

Figure S6. Temperature dependence of the Seebeck coefficient.

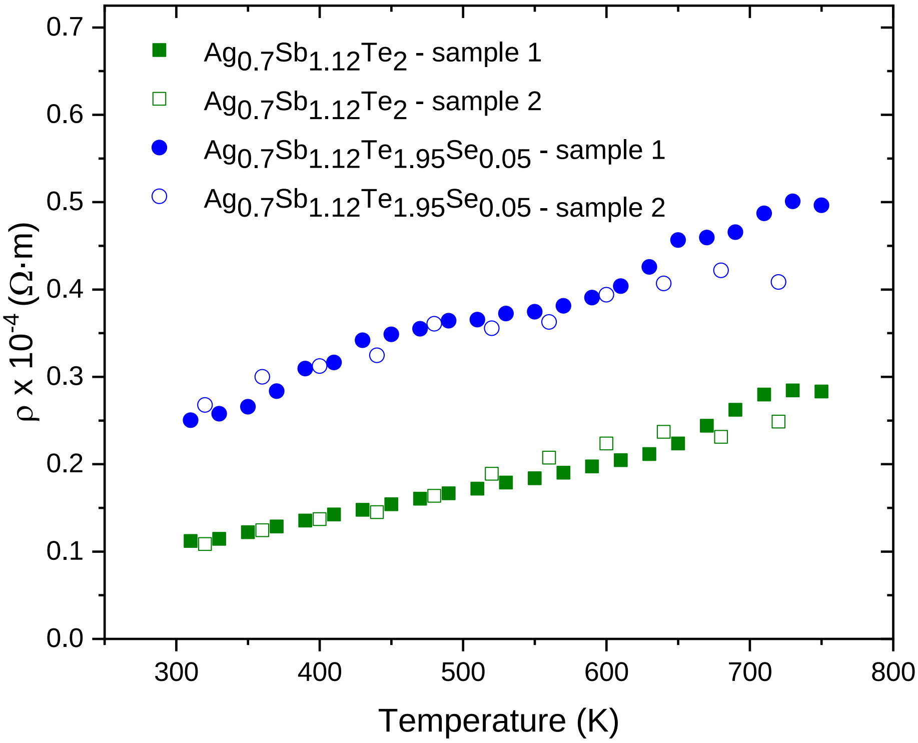

Figure S7. Temperature dependence of the electrical resistivity.

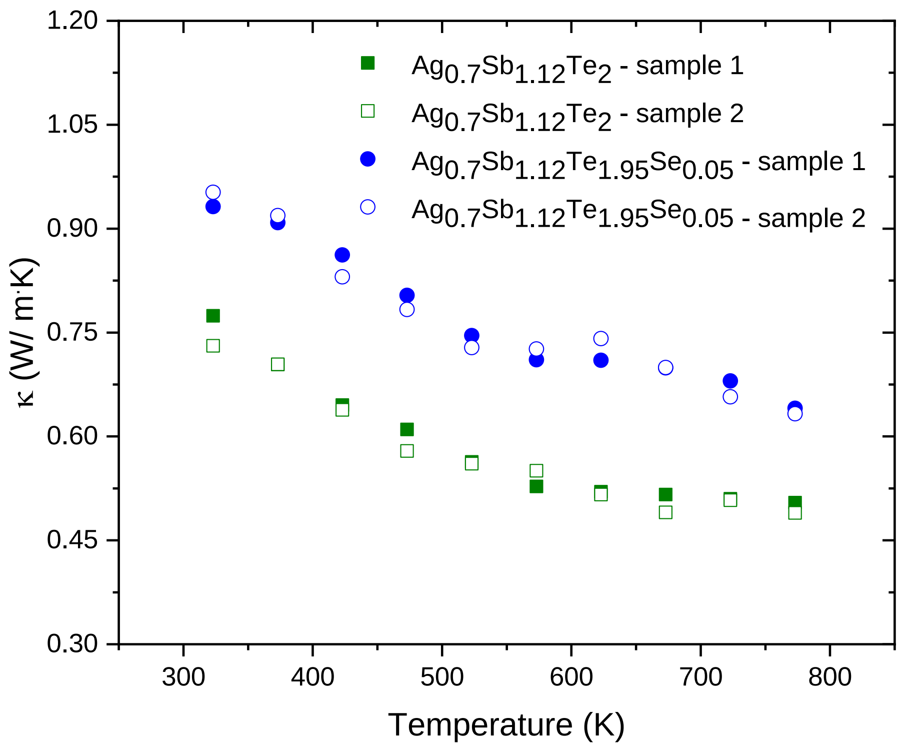

Figure S8. Temperature dependence of the thermal conductivity.
